# Fiber Fabry–Perot Sensor Based on Ion-Imprinted Sodium Alginate/Graphene Oxide Hydrogel for Copper Ion Detection Using Vernier Effect

**DOI:** 10.3390/s25030920

**Published:** 2025-02-03

**Authors:** Ning Wang, Shiqi Liu, Liang Xu, Longjiao Wang, Ming He, Chuanjie Lei, Linyufan Xiao

**Affiliations:** College of Science, China University of Petroleum (East China), No. 66 Changjiang West Road, Qingdao 266580, China; s22090002@s.upc.edu.cn (S.L.); z22090009@s.upc.edu.cn (L.X.); s23090002@s.upc.edu.cn (L.W.); z23090020@s.upc.edu.cn (M.H.); s23090001@s.upc.edu.cn (C.L.); z24090012@s.upc.edu.cn (L.X.)

**Keywords:** copper ions, optic fiber sensing, optical Vernier, selectivity

## Abstract

This work proposes an optical fiber copper ion sensor, which is fabricated by an ion-imprinted sodium alginate/graphene oxide (SA/GO) hydrogel and single-mode fiber (SMF). This sensing Fabry–Perot Interferometer (FPI) achieves −1.98 nm/(mg/L) sensitivity with 0.998 linearity. To achieve higher sensitivity, we add a reference FPI to create a Vernier effect. We achieve 19.58 nm/mg/L sensitivity and 0.989 linearity at a concentration range of 0 mg/L–1.4 mg/L. It was 9.9 times higher than that of a single-sensing FPI. The experimental results also demonstrate that when the FSR values of two FPIs are closer, the higher response sensitivity is achieved. The sensor also has good measurement repeatability and dynamic response. In addition, the experimental results of response selectivity show that its response sensitivity to copper ions is significantly higher than other six types of ions, including iron ions, lead ions, magnesium ions, manganese ion, zinc ions, chromium ions. The copper ion is also mixed with six types of ions to deeply investigate the response selectivity. Good response selectivity and cross-responding are demonstrated by experimental results.

## 1. Introduction

Copper ion is a kind of heavy metal ion frequently found in industrial wastewater from chemical industries, dyeing, electroplating, non-ferrous metal mining, and electronic material cleaning processes [1]. The discharge of wastewater containing high levels of copper has a profound impact on the soil [2] and water resources [3,4] essential for human survival. Excessive intake of copper ions can also cause damage to human health [5,6,7], including kidney damage, liver necrosis, hemolysis, and brain tissue changes.

In the drinking water quality guidelines of the World Health Organization, the maximum allowable concentration of copper ions is 2 mg/L. According to the drinking water standards of the United States Environmental Protection Agency, the maximum allowable concentration of copper is 1.3 mg/L. In China, drinking water hygiene standards state the copper concentration in tap water should not exceed 1 mg/L.

Therefore, the measurement of copper ion concentrations is crucial to ensure human health and prevent ecological damage from copper pollution [8]. Common methods for measuring copper ions include fluorescence labeling [9,10], atomic absorption spectroscopy [11,12], and electrochemical techniques [13,14,15]. However, these methods generally have high detection costs, complex sample preparation, and lengthy detection times.

Compared to traditional sensors, optic fiber sensors [16] offer significant advantages such as electromagnetic interference resistance, high sensitivity, simple manufacturing processes, low cost, high-temperature endurance, and corrosion resistance [17]. These attributes make them highly potent for metal ions measurements. In 2024, Anuradha and colleagues [18] proposed a fiber multi-mode interference multi-channel transmission sensor based on the parallel combination of a single mode-claddingless-graded index mult-mode-single mode (SCGS) fiber structure and a single mode-claddingless-few mode-single mode (SCFS) fiber for simultaneous tracking of Cu^2+^ and Zn^2+^ metal ions. In one channel, an SCGS fiber structure combination is used to measure Cu^2+^ from 100 nM to 1000 nM with 4.347 × 10^−4^ nm/nM accuracy. In the other channel, an SCFS fiber combination is used to measure Zn^2+^ in the same range with 4.358 × 10^−4^ nm/nM sensitivity. In 2023, Yuanli Xu and colleagues [19] developed a surface plasmon resonance (SPR) sensor based on a tri-core fiber (TCF) coated with a film composed by mercaptoundecanoic acid (MUA), non-ionic polyacrylamide (NPAM), and zinc oxide (ZnO). This sensor was used to measure chromium ions (Cr^3+^) in water. In the same year, Weixiang Yuan and colleagues [20] fabricated a sensor-combined no-core fiber with a multi-mode fiber coated with a chitosan (CS)/α-Fe_2_O_3_ nanocomposite. The results showed a maximum sensitivity of 456.69 nm/μM for Cu^2+^ concentrations measurement from 0 to 1 μM, with 0.989 linearity. In 2022, Yi Cai [21] proposed an optic fiber fluorescence sensor with carbon quantum dots for Fe^3+^ measurement in the range of 0–250 μM. However, the reported sensors usually have low response sensitivities, complex structures, and high manufacturing costs. Furthermore, there are few reports on the application of optic fiber copper ion sensing.

Fabry–Perot optic fiber sensors have been developed over many years for the detection of many parameters such as temperature, pressure, strain, and so on. As a well-known sensor type, it has attracted many researchers in recent years because of its high performance, simple structure, and diverse design. In 2024, Yongxing Guo and colleagues [22] developed an optical fiber bubble Fabry–Perot Interferometer. The fabrication process involved welding a multi-mode fiber (MMF) to a single-mode fiber (SMF), followed by subjecting the end of the MMF to hydrofluoric acid (HF) corrosion and discharge treatments. Experimental results show that the sensor has a pressure sensitivity of −1 × 10^−1^ dBm/MPa and a temperature sensitivity of 1.52 × 10^−2^ dBm/°C. In 2023, Zikang Yin and colleagues [23] proposed a highly sensitive fiber optic Fabry–Perot temperature sensor based on polydimethylsiloxane (PDMS) filling of an air cavity. The sensor consists of a capillary quartz tube, an optical fiber, and the temperature-sensitive material PDMS. In the temperature range of 5 °C to 40 °C, its sensitivity is no less than 7.34611 nm/°C. In particular, Fabry–Perot optic fiber sensors can be flexibly combined with a lot of sensitive materials to obtain various sensors, which can be used in environmental monitoring [24]. Considering the present situation of few application report about optic fiber copper ion sensing, the Fabry–Perot optic fiber sensor is a good choice to develop an optic fiber copper ion sensor. However, the sensitivity improvement of single FPI is often limited by structural and material properties. For simple and rapid sensitivity improvement, the optic Vernier effect is a good method.

The optic Vernier effect utilizes the minute scale differences between the main and auxiliary scales to refine measurements [25]. Sensor sensitivity can be improved by the Vernier effect [26]. In 2024, Yitong Li and others [27] proposed a highly sensitive optical fiber dissolved ammonia sensor composed of a sensing Fabry–Perot Interferometer (S-FPI) and a reference Fabry–Perot Interferometer (R-FPI) in parallel. Experimental results indicate that the sensor sensitivity is 0.34 nm/ppm in the dissolved ammonia range of 5–40 ppm, which is approximately 9.1 times higher than single S-FPI. In 2021, Xulong Jia and colleagues [28] proposed an optic fiber temperature sensor that combined a Sagnac interferometer and a Mach–Zehnder interferometer to achieve the Vernier effect. The experimental results showed that the temperature sensitivity of a single FSI was only 1.68 nm/°C, but the cascaded system with the Vernier effect could amplify it to 14.30 nm/°C. Although the Vernier effect has been effectively applied in optic fiber sensors for measuring refractive index [29,30], temperature [31], humidity [32], and air pressure [33], its application in metal ion concentration measurement is seldom reported.

To achieve an optic fiber copper ion sensor with good performance, which includes high sensitivity, a linear response, good repeatability, obvious selectivity, a fast dynamic response, easy fabrication, and a low cost, we fabricated an optical fiber Fabry–Perot copper ion sensor through a sodium alginate/graphene oxide hydrogel and an ordinary optic fiber, and we improved its sensitivity by the Vernier effect. The experimental results showed that the sensor has good response performance, which is very helpful to develop an optic fiber copper ion sensor with good performance.

## 2. Materials and Methods

### 2.1. The Working Principle of Sensor

**Figure 1:**
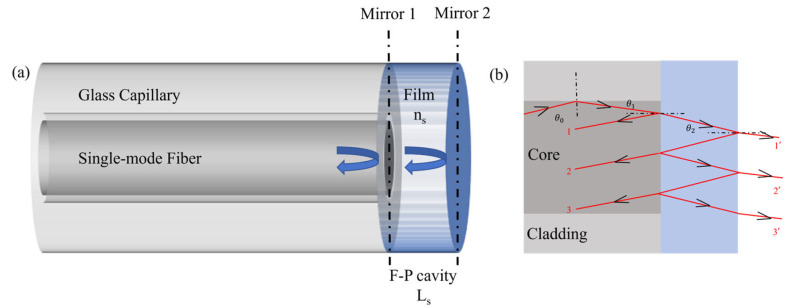
(**a**) Sensor structure diagram; (**b**) optical transmission model in the sensor.

The sensor employs a Fabry–Perot Interferometer (FPI) structure as shown in Figure 1a, which is composed of single mode optical fiber and sensitive film. Mirror 1 of FPI is the reflective surface between the single-mode optical fiber and the sensitive film. Mirror 2 is the reflective surface between the sensitive film and the external environment. The interference principle is related to the transmission light from the optical fiber to the sensitive film, as shown in Figure 1b. The Fabry–Perot Interference can be considered as a dual-beam interference, and its reflected light intensity can be expressed as follows [34]:


(1) $$I_{s}=I_{s1}+I_{s2}+2\sqrt{I_{s1}I_{s2}}\cos\left(\frac{4\pi n_{s}L_{s}}{\lambda }+\varphi_{0}\right),$$

In Equation (1), *I_s1_* and *I_s2_* represent the reflected light intensity of Mirror 1 and Mirror 2, *n_s_* and *L_s_* represent the refractive index and thickness of the hydrogel, and *φ_0_* is the initial phase.

When the intensity, Is, reaches its maximum value, the peak wavelength of the reflection spectrum can be expressed as follows [35]:


(2) $$\lambda_{m}=\frac{2n_{s}L_{s}}{m},m=0,1,2,3,\ldots$$

In Equation (2), m is the ordinal number of interference peaks. According to the above equations, the Free Spectral Range (FSR) can be expressed as follows:


(3) $${\mathrm{FSR}}_{s}=\frac{{\left(\lambda_{m}^{\prime }\right)}^{2}}{{\mathrm{OPD}}_{s}}=\frac{{\left(\lambda_{m}^{\prime }\right)}^{2}}{2n_{s}L_{s}},$$

In Equation (3), OPDs is the optical path difference between the two reflection lights in Figure 1a. In the experiments, the sensor is inserted into copper ion solutions with different concentrations. When the concentration of copper ions changes, the corresponding spectrum peak wavelength change is expressed as follows:


(4) $${∆\lambda }_{s}=\frac{2\lambda_{m}}{{\mathrm{OPD}}_{s}}\left(\frac{\partial n_{s}}{\partial \left(C_{Cu^{2+}}\right)}L_{s}+n_{s}\frac{\partial L_{s}}{\partial \left(C_{Cu^{2+}}\right)}\right),$$

Among them, m is an integer. $C_{Cu^{2+}}$ is the concentration of copper ions. When the sensor is inserted into the copper ion solutions, the refractive index and thickness of the hydrogel change. This leads to the spectrum peak wavelength change $\left({∆\lambda }_{s}\right)$. So, the peak wavelength tracking of interference spectrum can be used to evaluate the concentration change of copper ions.

**Figure 2:**
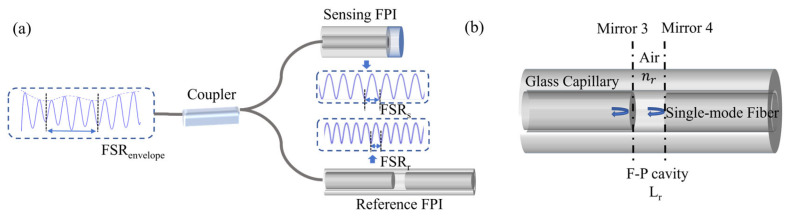
(**a**) Illustration of the Vernier effect; (**b**) enlarged structural diagram of the reference FPI.

To achieve higher sensitivity, we constructed an optical Vernier by adding a reference FPI, as Figure 2a shows. The cavity of reference FPI is empty, as Figure 2b shows. The design of an empty cavity is for the convenience of adjustment. It is important to achieve a good Vernier effect. According to Equation (2), the spectrum peak wavelength of reference FPI can be expressed as follows:


(5) $$\lambda_{m}^{\prime }=\frac{2n_{r}L_{r}}{m},m=0,1,2,3,\ldots$$

In the equation, *n_r_* is the refractive index of the air and *L_r_* is the length of the air cavity. According to the above equation, it can be seen that its FSR can be expressed as follows:

(6) $${\mathrm{FSR}}_{s}=\frac{{\left(\lambda_{m}^{\prime }\right)}^{2}}{{\mathrm{OPD}}_{r}}=\frac{{\left(\lambda_{m}^{\prime }\right)}^{2}}{2n_{r}L_{r}},$$

where OPD_r_ is the optical path difference between the two reflection lights in reference FPI.

When two FSR values of the sensing FPI and the reference FPI are close but not equal, the dual-cavity structure will generate an optical Vernier effect, forming a periodic envelope. This waveform, as Figure 2 shows, is the superposition of two FPI spectra. So, this superimposed spectrum is also shifted when the sensing FPI is inserted in different solutions. The FSR of the envelope is as follows [36]:


(7) $${\mathrm{FSR}}_{\mathrm{envelope}}=\frac{\mathrm{FS}{\mathrm{FSR}}_{r}{\mathrm{FSR}}_{s}}{\left|{\mathrm{FSR}}_{r}-{\mathrm{FSR}}_{s}\right|},$$

The FSR_envelope_ can be described as follows [37]:

(8) $${\mathrm{FSR}}_{\mathrm{envelope}}=\frac{{\mathrm{FSR}}_{s}{\mathrm{FSR}}_{s}}{\left|\mathrm{FS}R_{r}-FSR_{s}\right|}=\left|\frac{\lambda_{1}\lambda_{2}}{{OPD}_{r}-{OPD}_{s}}\right|,$$

where FSRs is the FSR of sensing FPI, and FSRr is the FSR of reference FPI. $\lambda_{1}$ and $\lambda_{2}$ are the center wavelengths of interference spectrum of two FPI.

The peak wavelength shift in the envelope wave is as follows [38]:


(9) $${∆\lambda }_{envelope}=\frac{2\lambda_{m}}{{\mathrm{OPD}}_{r}-{OPD}_{s}}\left(\frac{\partial n_{s}}{\partial \left(C_{Cu^{2+}}\right)}L_{s}+n_{s}\frac{\partial L_{s}}{\partial \left(C_{Cu^{2+}}\right)}\right),$$

According to Equation (9), when the difference value of *OPD_r_* and *OPD_s_* is small, the value of ${∆\lambda }_{envelope}$ is big. This means the response sensitivity is higher.

An important characteristic of the Vernier effect is the Magnification Factor (M-factor) [39]. Although there are two definitions for the M-factor, both definitions provide approximately the same result [25]. Because the sensitivity of the Vernier effect is higher than the sensitivity of a single-sensing interferometer, the first definition of the M-factor is the ratio between the sensitivity of the Vernier envelope (*S_envelope_*) and the sensitivity of single-sensing interferometer (*S_s_*):

(10) $$M=\frac{S_{envelope}}{S_{s}},$$

Another definition of M-factor is the ratio of the FSR of the Vernier envelope to the FSR of single-sensing interferometer. It is expressed as follows:


(11) $$M=\frac{{FSR}_{envelope}}{{FSR}_{s}}=\frac{{FSR}_{r}}{\left|{FSR}_{r}-{FSR}_{s}\right|}$$

So, the M-factor value is larger when two FSR values are closer.

### 2.2. Sensor Fabrication and Sensing Experiment System

The designed sensor uses standard single-mode optical fibers (SMF-G657A1A, Yangtze Optical Fiber and Cable Joint Stock Limited Company, Yichang, China), micro-capillaries (DE-M 22, HIRSCHMANN, Neckartenzlingen, Germany), an optical fiber coupler (FC/APC, Shenzhen Aokangda Optoelectronics Co., Ltd., Shenzhen, China), and a laboratory-synthesized sodium alginate/graphene oxide (SA/GO) hydrogel [40] that is sensitive to copper ions. The materials for hydrogel preparation include graphene oxide (GO, Changzhou Sixth Element Materials Technology Co., Ltd., Changzhou, China), sodium alginate (SA, Shanghai Aladdin Biochemical Technology Co., Ltd., Shanghai, China), calcium chloride (Sinopharm Chemical Reagent Co., Ltd., Beijing, China), and Cu(NO_3_)_2_ (Shanghai Macklin Biochemical Co., Ltd., Shanghai, China).

**Figure 3:**
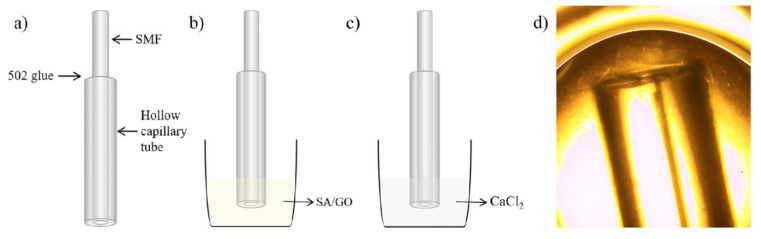
The manufacturing process of sensing FPI: (**a**) Probe fixation; (**b**) coating sensitive materials; (**c**) calcium chloride solidification; (**d**) sensor S_1_ under microscope.

The sensor fabrication process is shown in Figure 3. Firstly, we peel off the fiber coating layer, clean the optical fiber with alcohol wiping paper, and use a fiber cutting knife to cut the single-mode optical fiber to achieve a flat-end surface. Then, we insert the fiber into a capillary tube, which has a 0.14 mm inner diameter and 0.57 mm outer diameter. By microscope, we adjusted the fiber position until the fiber end face is aligned flush with the capillary end face. Then, 502 adhesives are used to fix the single-mode fiber and capillary at the tail end. The prepared probe is dipped into the sensitive materials (SA/GO hydrogel) to achieve a sensitive film in the end, and this was placed in a 5% mass fraction CaCl_2_ solution [41,42] to stand for 3 h. Then, we transfer the sensor to deionized water and conduct elution for 12 h. The sensing FPI is denoted as sensor 1 (S_1_).

**Figure 4:**
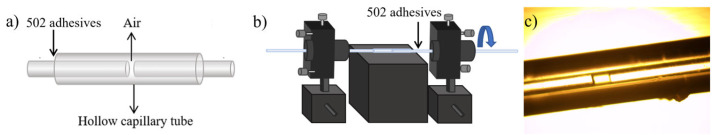
The manufacturing process of reference FPI: (**a**) Probe fixation; (**b**) cavity length adjustment; (**c**) reference FPI under microscope.

The reference FPI fabrication needs two fibers and a capillary tube. We cut two fibers as before and insert them into the capillary from two ends. Then, we fix them in an optical fiber adjustment rack and adjust two fibers until a good spectrum is achieved by the SM125 optic fiber sensing analyzer (Micron Optics Company, America). In total, 502 adhesives are also used to fix the fiber and capillary together. The real photo from the microscope is shown in Figure 4. The reference FPI is achieved. The use of a reference FPI in transmission is to more flexibly adjust the cavity length of the reference FPI because of empty cavity. This is very important to improve the sensitivity of the sensor because we must achieve a suitable FSR value of the reference FPI for the Vernier effect.

Finally, we use an optical fiber coupler to connect the sensing FPI and the reference FPI. The sensor system with the Vernier effect is finished. In the whole fabrication process, two FPIs are both connected to the SM125 analyzer to monitor the spectrum.

For sensitive materials, we used an SA/GO hydrogel as the choice of sensing FPI because of its easy preparation, good absorption ability, and good adhesive ability to the fiber and the capillary. Sodium alginate and graphene oxide contain a large number of oxygen-containing functional groups, such as a carboxyl group and a hydroxyl group. They can form cross-linking bonds with metal cations. Meanwhile, graphene oxide has large specific surface area, providing enough sites for Cu^2+^ adsorption.

The SA/GO hydrogel is easy prepare in experiments. Firstly, we mixed 0.32 g graphene oxide and 40 mL deionized water with a breaker. Then, we stirred it magnetically for 1 h and sonicated it for 1 h to obtain a uniformly dispersed suspension of graphene oxide. Secondly, we mixed 0.8 g sodium alginate and 35 mL deionized water with another beaker and stirred it magnetically for 1 h. Then, we added 80 mg graphene oxide suspension from breaker 1 and stirred them for 2 h to obtain SA/GO sol.

To improve the selectivity of sensitive materials towards copper ions, the ion imprinting method was used by adding 5 mL Cu (NO_3_)_2_ solution with 1 g/L concentration to the SA/GO solution, followed by stirring for 20 min. Then, the sensing FPI sensor is dipped with some sol and placed in 5% CaCl_2_ solution for 3 h to solidify and then placed in deionized water to elute excess copper ions. Finally, we obtain an ion-imprinted SA/GO hydrogel film at the sensor end.

**Figure 5:**
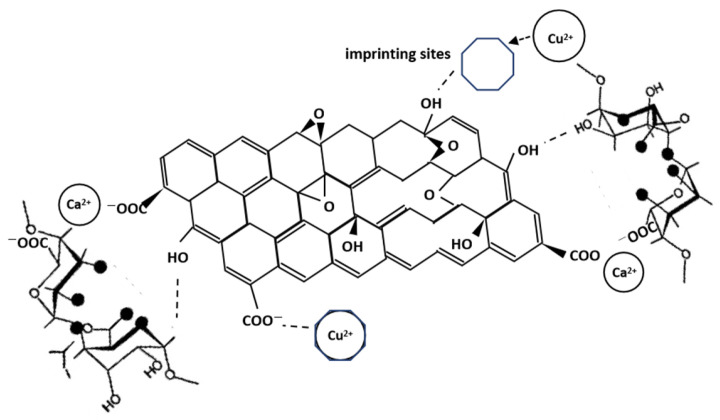
Adsorption mechanism of copper ions.

In the ion imprinting process, Cu^2+^ was added as a template ion to the mixed SA/GO solution. It formed a complex with sodium alginate and graphene oxide through electrostatic adsorption. CaCl_2_ is simply used as a crosslinking agent for the polymerization reaction in the gel process. After the copper ions were eluted by ionic water, SA/GO hydrogel obtains many holes (i.e., imprinting sites) that are highly matched with the target copper ions in shape, size, and coordination environment. This special structure enables hydrogel to achieve good efficient adsorption and selectivity of Cu^2+^ by the imprinting sites. The adsorption mechanism of copper ions is shown in Figure 5.

**Figure 6:**
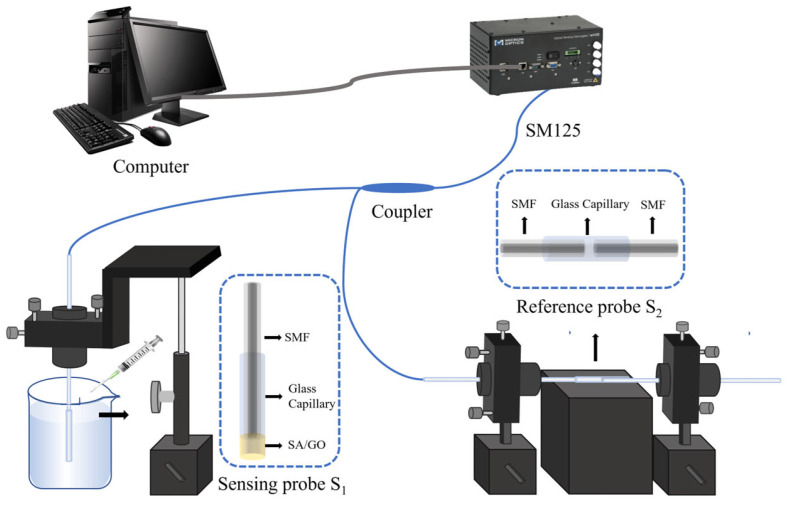
Experimental setup for copper ion measurement.

Figure 6 shows the schematic diagram of the copper ion sensing experiment system. The experimental setup consists of two sections: module control and testing area. The module control area is composed of Micron Optics SM125 optic fiber sensing analyzer and a computer equipped with the MOI software (1.0.38e). The test area includes an optic fiber copper ion sensing head and the test solution. The Vernier effect sensing structure is constructed by paralleling the sensing FPI and the reference FPI with a 50:50 1 × 2 optic fiber coupler. In the experiment, the SM125 optic fiber sensing analyzer serves as light source and spectrometer, with 18 mW output power, and 1510–1590 nm wavelength range with 0.001 nm accuracy. The laboratory temperature is maintained at 20 °C in experiments. The test solution used is a copper nitrate solution. We can adjust the Cu^2+^ concentration to obtain different test samples. The computer equipped with the MOI software is used to collect the spectrum data. The data of spectral shifts are focused for further analysis.

## 3. Results

### 3.1. Sensor Stability Test

**Figure 7:**
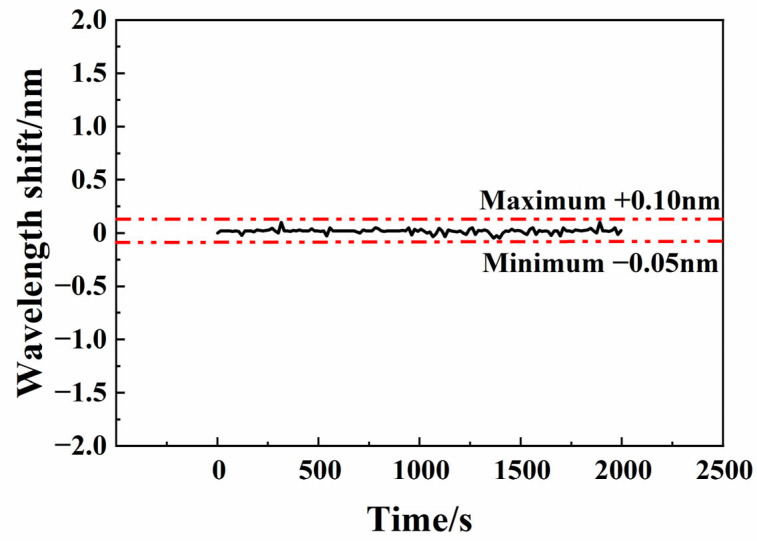
Wavelength drift over a long period of time.

We fix the fabricated sensor in the beaker with a copper ion solution of 0.2 mg/L and adjust it to be immersed in solution. To investigate the response stability, the sensor response spectrum is continually monitored for 2000 s. The peak wavelength shift in interference spectrum is shown in Figure 7. According to experimental data, the maximum peak wavelength shift is 0.10 nm, which shows good spectrum responding stability.

### 3.2. Cu^2+^ Concentration Response Experiments of Single=Sensing FPI Sensor

In Cu^2+^ concentration response experiments, different concentrations of copper ion solutions were measured at 0, 0.2, 0.4, 0.6, 0.8, 1.0, 1.2, and 1.4 mg/L. The interference spectrum changed at different concentrations of copper ion solutions. The spectrum data were continually monitored and recorded by MOI SM125. In experiments, when the concentration is more than 1.4 mg/L, the spectrum response is still obvious. However, the spectrum peak tracking shifts beyond the measurement wavelength range of SM125 (1510–1590 nm). So, we repeated the response experiments in different solutions up until 1.4 mg/L.

**Figure 8:**
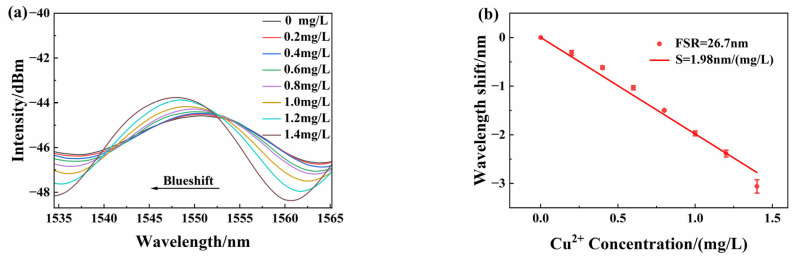
(**a**) Wavelength shifts detected by sensor S_1_ at different copper ion concentrations; (**b**) peak wavelength linear characteristics.

Figure 8 presents the response results of sensor S_1_ (No sensor S_2_). The FSR of the sensor is 26.7 nm. Because the cavity length measurement error under the microscope is very big considering the real film surface situation, we use the FSR value as a sensor parameter instead of cavity length. As shown in Figure 8a, with increased concentration of copper ions, the reflected spectrum shifted noticeably toward shorter wavelengths (blue shift). The peak wavelength shift is related to refractive index change and film thickness change as Equation (4) shows, because the SA/GO hydrogel absorbed the Cu^2+^ of solutions. When the concentration is 1.4 mg/L, the wavelength shift is approximately 2.9 nm. By tracking the typical spectrum peak near 1550 nm, the peak wavelength values at various concentrations were measured, as shown in Figure 8b. The slope obtained from the linear fitting represents the sensor response sensitivity (*S_s_*), approximately 1.98 nm/(mg/L). The linearity is 0.998, which indicates a good linear relationship between peak wavelength shift and copper ion concentration. High sensitivity and good linear responses were demonstrated.

### 3.3. Sensor Response Selectivity Improvement

**Figure 9:**
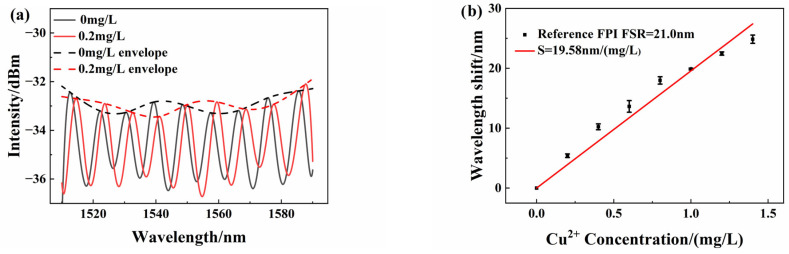
(**a**) Wavelength shifts as detected by the parallel sensor at different copper ion concentrations; (**b**) peak wavelength linear characteristics.

As mentioned earlier, the Vernier effect can improve sensitivity. As shown in Figure 6, the Vernier effect sensing structure is constructed by paralleling the sensing FPI and the reference FPI with a 50:50 1 × 2 optic fiber coupler. The sensing FPI S_1_ is placed inside the copper ion solution. The reference FPI S_2_ is not in solution, which is adjusted by the fiber optic adjustment rack. The FSRs of sensing FPI S_1_ and reference FPI S_2_ are 26.7 nm and 21.0 nm, respectively. The response spectrum of the Vernier effect sensing structure is measured as Figure 9a shows. The measurement is repeated at different concentrations of solutions. Figure 9a illustrates the spectrum shift when the copper ion concentration changed from 0 mg/L to 0.2 mg/L. The dashed line is the envelope of the response spectrum. For the Vernier effect measurement method, the spectrum envelope is focused as an effective response spectrum. So, the peak wavelength shift value of the envelope is used to evaluate the response wavelength shift value of spectrum peak tracking. According to this method, peak wavelengths of the response spectrum at different concentrations are measured. Figure 9b is the peak wavelength shift value with different Cu^2+^ concentration. The response sensitivity is approximately 19.58 nm/(mg/L) by linear fit, with 0.989 linearity. The experimental results indicate that the Vernier sensing structure also has high higher sensitivity and a good linear response. Compared to single-sensor experiments before, the Vernier sensing structure shows a 9.9-fold increase in sensitivity.

**Figure 10:**
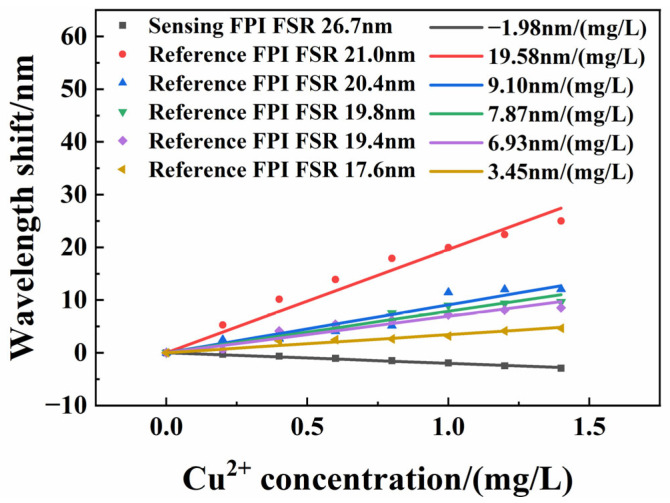
Wavelength shift as recorded by the envelope with different reference FPIs and the same sensing FPI.

As Equations (8) and (11) show, the FSR values of the sensing FPI and the reference FPI directly influenced the degree of sensitivity improvement. To further investigate the response characteristics of the Vernier sensing structure, under the premise of ensuring the cursor effect, we test the same sensing probe with five reference FPIs that have different FSR values. The FSR values of five reference FPIs were 21.0, 20.4, 19.8, 19.4, and 17.6 nm, respectively, with a concentration test range of 0–1.4 mg/L. The FSR of the sensing FPI is 26.7 nm. The results are shown in Figure 10. The sensitivities of the Vernier sensing structure were 19.58, 9.10, 7.87, 6.93, and 3.45 nm/(mg/L), respectively. We compared these results of the Vernier sensing structure to foregoing a single sensor with 1.98 nm/(mg/L) sensitivity. The sensitivity magnification is 9.90, 4.59, 3.97, 3.50, and 1.74, respectively. It is evident that higher response sensitivity is achieved when the FSR values of two FPIs are closer. According to Equation (11), when two FSR values of two FPIs are closer, the M-factor is higher. According to Equation (10), the sensor sensitivity, S_envelope_, is higher. This is also consistent with the discussion of reference [25].

### 3.4. Sensor Dynamic Response

**Figure 11:**
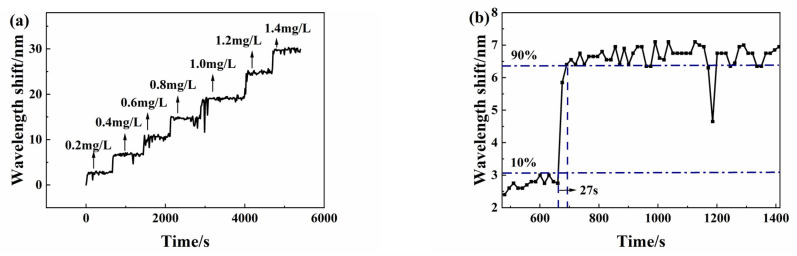
(**a**) The dynamic response of the sensor at different copper ion concentrations; (**b**) the enlarged view of typical dynamic response results.

For investigation of the dynamic response, the Micron Optics SM125 optic fiber sensing analyzer is set to automatic acquisition mode with 30 Hz acquisition frequency for continuous measurement of spectral data. When the sensor is placed in the next copper ion solution, the spectrum peak wavelength change is automatically recorded in a whole dynamic measurement process. The test solution concentration remains from 0 mg/L to 1.4 mg/L. In spectral data processing, the peak wavelength shift values of the envelope spectrum are recorded. Finally, we obtain the dynamic response experimental curve as Figure 11a shows. The step curves show an obvious dynamic response of the sensor.

In order to observe the response time more clearly, the typical dynamic response experimental results in Figure 11a are enlarged, as shown in Figure 11b. When the copper ion concentration increases from 0.2 mg/L to 0.4 mg/L, the response time is 27 s.

### 3.5. Sensor Response Repeatability

**Figure 12:**
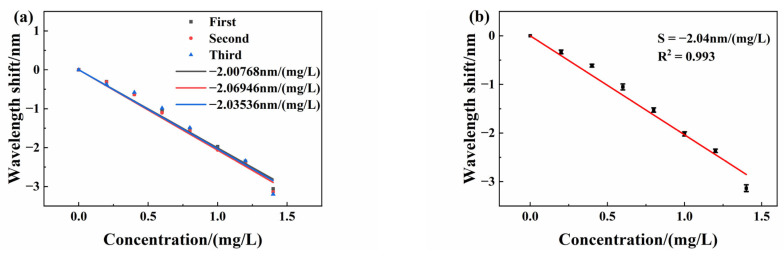
(**a**) Wavelength shift in a sensor spectrum in repeatability experiments; (**b**) error bar fitting curve with different concentrations.

The sensor repeatability is very important for evaluating its performance and reliability, which reflects the degree of inconsistency when the sensor is tested multiple times across the full range. We placed the sensing FPI in different test solutions from 0 mg/L to 1.4 mg/L for measurement, which forms one test. Then, we soak the sensing FPI in deionized water to desorb copper ions. After the interference spectrum stabilizes, we place the sensing FPI again in the test solution and repeat the former experiments. We test the same sensor three times from 0 mg/L to 1.4 mg/L. All spectrum data are recorded in three tests. The experimental curves of the relationship between the interference spectrum drift and copper ion concentration are shown in Figure 12a. The error bars are also shown in Figure 12. Figure 12b is the error bar fitting curve with different concentrations. Typical standard deviation is approximately 0.0425 mg/L at a copper ion concentration of 1 mg/L. The results show good repeatability of the sensor.

### 3.6. Sensor Response Selectivity

In real applications, many metal ions are mixed together in polluted water, which can interfere with the Cu^2+^ measurement. For effectively measuring Cu^2+^ concentration, the sensor selectivity that investigates for copper ions is important. For this purpose, the response of this sensor to six other metal ions, such as lead, magnesium, zinc, manganese, iron, and chromium, was tested. Finally, we obtained seven kinds of ion response results of the same sensor. The seven kinds of ion solutions are all 0.2 mg/L. At each test, the sensing sensor is inserted and transferred from a 0 mg/L to 0.2 mg/L solution. The peak wavelength of the interference spectrum will also shift like former experiments.

**Figure 13:**
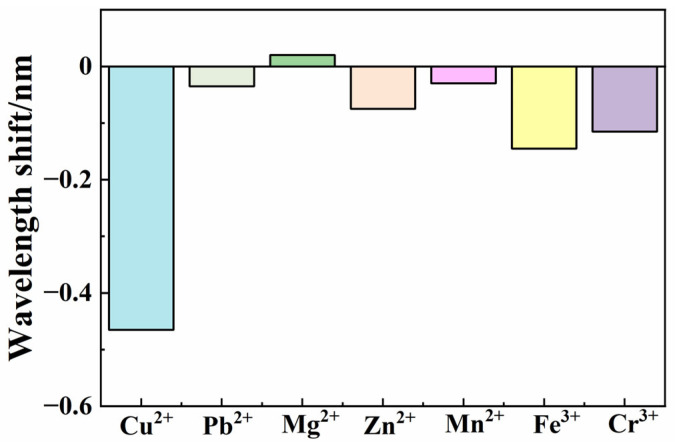
Wavelength shift in the spectrum to different metal ions.

We recorded all spectral data and analyzed the peak wavelength shifts to different metal ions. Figure 13 shows the wavelength shift values to all seven kinds of metal ions. The response wavelength shifts are 0.465 nm (Cu^2+^), 0.035 nm (Pb^2+^), −0.02 nm (Mg^2+^), 0.075 nm (Zn^2+^), 0.03 nm (Mn^2+^), 0.145 nm (Fe^3+^), and 0.115 nm (Cr^3+^). The wavelength shift in relation to copper ion responses is significantly greater than that of other metal ions. The big difference in sensitivity showed good selectivity of copper ions responses. This is very helpful to solve cross-responding problems.

The results also show the opposite wavelength shift direction of magnesium ions compared to other ions. The spectral redshift phenomenon in the magnesium ion solution is related to the characteristics of hydrogel. With an increase in magnesium ion concentration, the expansion degree of hydrogel increases, which leads to an increase in the cavity length of the sensor. Due to the relatively low charge density of magnesium ions, the adsorption capacity of hydrogel for magnesium ions is small, so the refractive index of the sensitive material remains almost unchanged. Therefore, in a magnesium ion solution, an increase in cavity length will cause a redshift in the interference spectrum.

However, when the sensor is placed in a solution of other metal ions, the sensitive material will adsorb these ions, resulting in an increase in refractive index. Although the hydrogel can also swell and cause an increase in cavity length, the refractive index change is more significant than that of magnesium ion solution. The FSR of the sensor increases in other metal ion solutions, indicating that the change in refractive index has a more significant impact on the interference spectrum, compared to the increase in cavity length caused by swelling, leading to blueshift in their interference spectrum.

**Figure 14:**
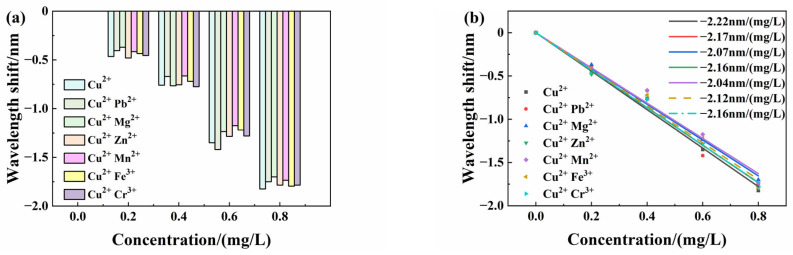
Response characteristics of the sensor under interference of other metal ions: (**a**) Wavelength drift of the sensor; (**b**) linear characteristics of sensors.

To further investigate the response selectivity to copper ions, copper ion solutions were mixed with other metal ion solutions. So, we obtained six kinds of mixed ion solutions. Then, the sensor was inserted in all kinds of mixed solutions to investigate the cross-responding ability. Each kind of mixed solution concentration was adjusted from 0 mg/L to 0.8 mg/L, with a 0.2 mg/L change step. Then, we tested the sensor response at different concentrations of each mixed ions solution. Figure 14a shows the peak wavelength shift in six kinds of mixed solution and a Cu^2+^ solution at different concentrations. Figure 14b is the linear fitting response curves.

The experimental results showed that the peak wavelength shifts to six kinds of mixed solutions are very close to non-mixed solutions, even if in different concentrations. The interference ions in mixed solutions, including lead, magnesium, zinc, manganese, iron, and chromium ions, only have a minor influence on the copper ion response sensitivity. So, the sensor has good response selectivity and cross-responding ability. Moreover, the linear response is still good in mixed solutions, like Figure 14b shows.

Good selectivity is attributed to the ion imprinting method in the preparation of the SA/GO hydrogel. During the hydrogel preparation, a certain amount of Cu^2+^ is added. After the sensor is placed in deionized water to wash away the excess Cu^2+^ for ion imprinting, many adsorption sites for Cu^2+^ in the hydrogel facilitates easier access of Cu^2+^. So, Cu^2+^ can be easily absorbed into the internal spaces of the SA/GO hydrogel, where they can interact with the functional groups.

## 4. Discussion

The traditional copper ion sensors include colorimetric sensors, fluorescent sensors, and electrochemical sensors. Table 1 shows the comparison between some reported copper ion sensors and our work. Compared with these sensors, the sensor of this paper exhibits superior performance. For colorimetric sensors [43], a complex measurement process is used, including sampling and adjusting the test solution to the appropriate pH value, dropping the test solution onto the test paper, letting it stand for color development, and then taking photos for image analysis. The measurement of our sensor is more convenient. The response time of some reported electrochemical sensors [44] is 200 s. The response time of a Schiff-based fluorescence sensor is about 2.5 min [45]. Our typical sensor has a shorter response time of about 27 s.

Compared with the sensor of reference [20], our sensor has a simple structure and larger measurement range. The optical fiber sensor of reference [46] has a wider response range, but the linear response is not good. It also has a complex and high-cost coating fabrication process with functionalized gold nanoparticles (Au NPs) containing 2′-mercaptobenzimidazole (MBI)/polyvinylpyrrolidone (PVP). Compared to the optical fiber plasmonic sensor of reference [47], the sensitive material of our work is easy to prepare and is low-cost. Compared to the hyperspectral sensing method [48], our sensor is easy to operate and does not require sample pre-treatment. Consequently, the sensor proposed in this paper is low-cost, easy to manufacture, and simple to operate, with high sensitivity, a good liner response, obvious selectivity, and a fast response. It has good application prospects in environmental monitoring and other fields.

## 5. Conclusions

This article investigated a copper ion sensor that uses ion-imprinted hydrogels and the Vernier effect by two fiber-optic Fabry–Perot Interferometers. It has high response sensitivity to copper ion concentrations in the range of 0 mg/L–1.4 mg/L. When the FSR of the sensing FPI is 26.7 nm and the FSR of the reference FPI is 21 nm, the sensor response sensitivity is 19.58 nm/(mg/L), with a linearity of 0.987, which is 9.9 times higher than that of a single-sensing FPI. It is verified that the sensor has a high response sensitivity and good linear response characteristics. In addition, the influence of FSR between the sensing FPI and the reference FPI on sensor sensitivity was studied. Higher response sensitivity is achieved with closer FSR values between two sensor interference spectra. The experimental results also showed that the sensor has a good spectrum response stability, fast dynamic response, and good repeatability. We also investigated the response selectivity of copper ions mixed with other ions, including lead, magnesium, zinc, manganese, iron, and chromium ions, respectively. The experimental results showed that the sensor has good response selectivity for copper ions. This sensor has the advantages of simple structure, low cost, and good performance, which has the potential to be applied in many fields such as environmental measurements.

## Figures and Tables

**Table 1:** Comparison with other copper ion sensors.

Reference	Type	Fabrication	Sensitive Materials	Concentration Range	Sensitivity	Linearity	Response Time
[43]	Colorimetric sensor	\	\	0.13 mg/L and above	\	\	\
[44]	Electrochemistry	Modified carbon paste electrode	\	4–200 ng/mL	\	0.998	200 s
[45]	Fluorescent sensor	\	Schiff base	0.56 µg/L and above	\	\	2.5 min
[20]	Transmission	MMF, SMF, NCF	CS/α-Fe_2_O_3_	0–1µM	456.69 nm/µM	0.998	\
[46]	Evanescent wave	Plastic optical fiber(POF), phototransistor	MBI/PVPAu NPs	100–1000 mg/L	0.335 mV/(mg/L)	0.88	\
[47]	SPR	MMF, SMF	Au@AgPt NSs	0.1 fM–10 pM	633%/RIU	0.998	\
[48]	Hyperspectral sensing	POF	\	100–1000 mg/L	\	\	\
Our work	Fabry–Perot interference	SMF	SA/GO	0–1.4 mg/L	19.58 nm/(mg/L)	0.989	27 s

## Data Availability

The data underlying the presented results are not publicly available but may be obtained from the authors upon reasonable request.
